# Ecdysone signaling regulates specification of neurons with a male-specific neurite in *Drosophila*

**DOI:** 10.1242/bio.029744

**Published:** 2018-02-15

**Authors:** Binglong Zhang, Kosei Sato, Daisuke Yamamoto

**Affiliations:** Division of Neurogenetics, Tohoku University Graduate School of Life Sciences, Sendai, 980-8577, Japan

**Keywords:** Sexual dimorphism, Courtship behavior, Circuit remodeling, Metamorphosis, The *fruitless* gene

## Abstract

Some mAL neurons in the male brain form the ipsilateral neurite (ILN[+]) in a manner dependent on FruBM, a male-specific transcription factor. FruBM represses *robo1* transcription, allowing the ILN to form. We found that the proportion of ILN[+]-mALs in all observed single cell clones dropped from ∼90% to ∼30% by changing the heat-shock timing for clone induction from 4-5 days after egg laying (AEL) to 6-7 days AEL, suggesting that the ILN[+]-mALs are produced predominantly by young neuroblasts. Upon EcR-A knockdown, ILN[+]-mALs were produced at a high rate (∼60%), even when heat shocked at 6-7 days AEL, yet EcR-B1 knockdown reduced the proportion of ILN[+]-mALs to ∼30%. Immunoprecipitation assays in S2 cells demonstrated that EcR-A and EcR-B1 form a complex with FruBM. *robo1* reporter transcription was repressed by FruBM and ecdysone counteracted FruBM. We suggest that ecdysone signaling modulates the FruBM action to produce an appropriate number of male-type neurons.

## Abstract (precis)

**Summary:** The insect molting hormone ecdysone determines whether a single neuron develops a sex-specific structure, through crosstalk with signaling elements in a pathway dedicated to the sex-fate determination.

## INTRODUCTION

It is widely thought that the sex-determination mechanism is distinctly different between vertebrates and insects; in vertebrates, systemic androgens and estrogens primarily determine the sex of an entire body (Sekido and Lovell-Badge, 2009), whereas in insects, every cell adopts a sexual fate according to its own chromosomal composition, without any involvement of androgens and estrogens (Salz, 2011). The only exception to this rule is the presence of a low level of 17-β-estradiol in silkworms (Ohnishi et al., 1985), which has been suggested to promote synthesis of vitellogenin, a female-specific protein (Shen et al., 2015). Rather than using them for sex determination, insects use steroids to realize their unique developmental strategy of molting, which allows the step-wise enlargement of the body size in accompaniment with radical renovations of internal and external structures, and even the induction of abrupt changes in physiology and behavior (Spindler et al., 2009). The major components of steroids that induce molting are systemic α-ecdysone, synthesized in the prothoracic gland (Karlson, 1996), and its derivative, 20-hydroxyecdysone (β-ecdysone). Ecdysones bind to a heterodimeric nuclear receptor composed of EcR and Ultraspiracle (Usp) proteins, thereby regulating the transcription of downstream genes that are hierarchically ordered to orchestrate a complex series of biological events, leading to molting (Hill et al., 2013). The EcR subunit has three isoforms, EcR-A, EcR-B1 and EcR-B2, each with distinct roles and expression patterns (Hara et al., 2013; Hill et al., 2013; Yamanaka et al., 2013).

Despite an exhaustive study of ecdysone actions related to molting, much less attention has been paid to the potential roles of ecdysones in sexual development (Schwedes and Carney, 2012). In particular, adult animals exhibit sexual dimorphisms in morphology, physiology and reproductive behavior, many of which develop, in holometabolous insects, around the pupal stage, when the ecdysone titer changes dynamically (De Loof, 2008; Truman, 2005). It is therefore likely that neural circuitries for sexually dimorphic behaviors displayed by adults are laid out during this developmental stage, under the control of ecdysone signaling (Ito et al., 2013). There is evidence that the ecdysone pathway directly contributes to neural remodeling via dendrite pruning (Awasaki et al., 2006; Williams and Truman, 2005), and to cell death during metamorphosis by cooperating with epigenetic factors including CREB-binding protein CBP, a histone acetyltransferase (HAT; Kirilly et al., 2011). This invites speculation that crosstalk between ecdysone signaling and the sex-determination pathway might provide a means for the organism to create sex differences in an otherwise unisexual neural circuitry (Ito et al., 2013).

The neural basis for sexual behavior has been extensively analyzed in a genetic model organism, *Drosophila melanogaster*, in which *fruitless* (*fru*) and *doublesex* (*dsx*), two major transcription factor genes with the sex-determination function, are key players in the construction of the sexually dimorphic circuitry underlying mating behavior (Dickson, 2008; Pavlou and Goodwin, 2013; Yamamoto and Koganezawa, 2013). Whereas *dsx* is widely involved in the development of sexual traits in a variety of tissues, *fru*-dependent sexual differentiation is strictly restricted to the nervous system (Dickson, 2008; Pavlou and Goodwin, 2013; Yamamoto and Koganezawa, 2013). Among the four promoters of the *fru* gene, the most distal promoter (the P1 promoter) is dedicated to sexual function (Ryner et al., 1996), producing multiple transcripts that are translated only in the male nervous system (Lee et al., 2000; Usui-Aoki et al., 2000). The male-specific proteins thus produced are collectively called FruM (where ‘M’ stands for male). FruM is composed of five isoforms, three of which (FruAM, FruBM and FruEM) have been demonstrated to contribute to neural sexual differences (Billeter et al., 2006; Neville et al., 2014; von Phillipsborn et al., 2014). FruAM, FruBM and FruEM share the N-terminal BTB domain, with the distinct zinc finger motifs at their C-terminus (Ryner et al., 1996; Usui-Aoki et al., 2000). The FruBM isoform recruits chromatin remodeling factors such as Bonus (Bon), Histone deacetylase 1 (HDAC1) and Heterochromatin protein 1a (HP1a) to target sites on the genome, thereby regulating the transcription of genes involved in sex-specific neural development (Ito et al., 2012). Thus, FruBM seems to use an epigenetic mechanism in neural sex fate induction, just as ecdysone signaling does (Sedkov et al., 2003) in neural remodeling during metamorphosis.

In this study, we show that EcR and FruBM indeed interact at the molecular level to produce an appropriate number of neurons that are equipped with the male-specific neurite. Based on this observation, we suggest that the insect molting hormone ecdysone modulates the transcriptional activities of FruBM to induce sex-specific neurobehavioral characteristics. Thus, ecdysone may exert a sex-specific function comparable to that of vertebrate sex steroids, when it operates through the ecdysone receptor complex containing FruBM as a constituent.

## RESULTS

### *EcR* is a genetic modifier of *fru*

**Fig. 1.**
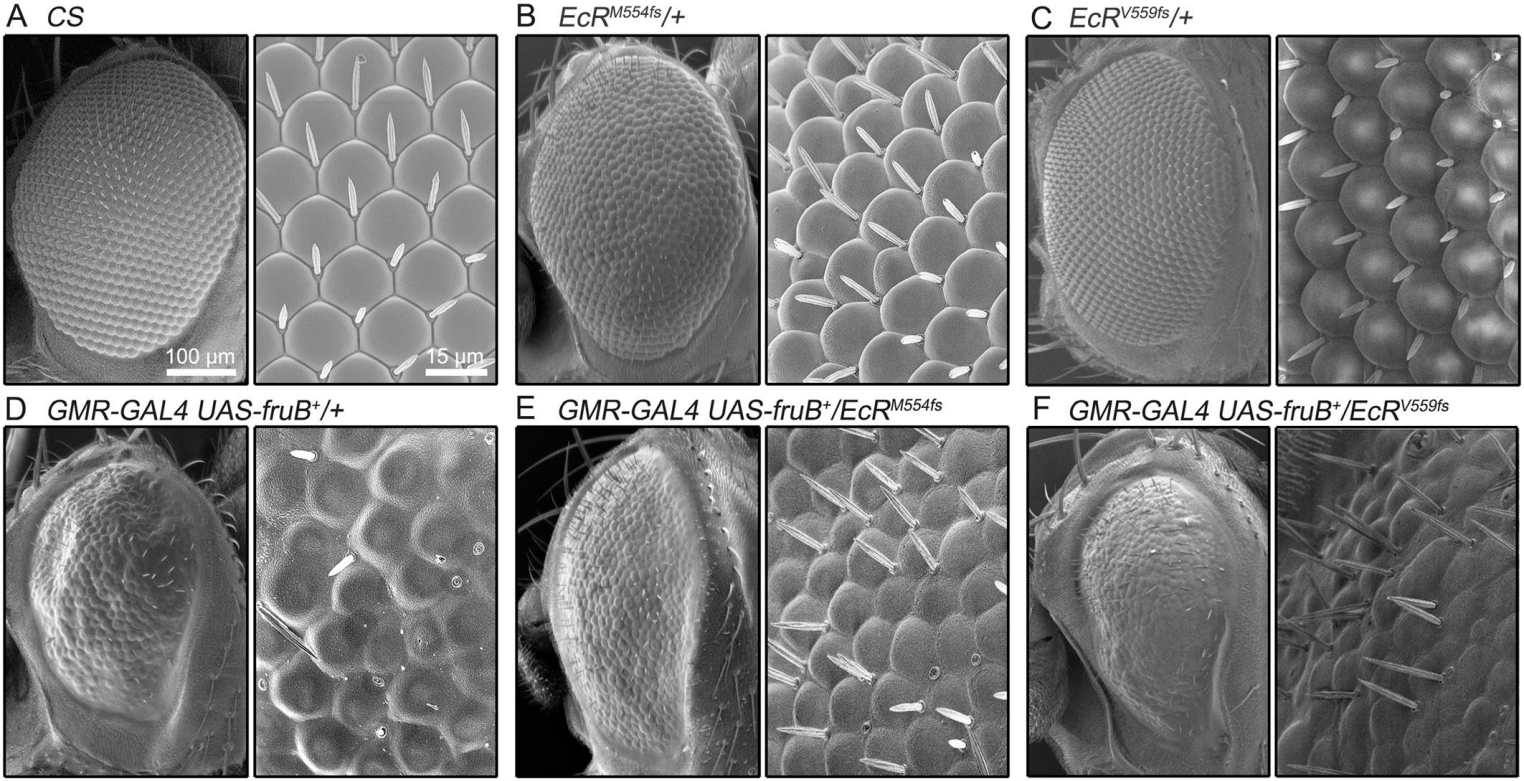
**The *fru* dominant eye phenotype is suppressed by a copy of loss-of-function *EcR* alleles.** (A-F) The compound eyes of a wild-type fly (A), an *EcR^M554fs^* heterozygote (B), an *EcR^V559fs^* heterozygote (C) and flies expressing *fruB^+^* via *GMR-GAL4* without (D) or with a copy of the *EcR^M554fs^* (E) or *EcR^V559fs^* (F) allele, shown at low (left-hand panels) and high (right-hand panels) magnifications. All eyes were from female flies. Scale bars: 100 µm (left) and 15 µm (right).

In searches for genes that interact with *fru*, we took advantage of a visible phenotype induced by overexpression of the normal form of FruB in the compound eye (Goto et al., 2011). In contrast to the regular array of ommatidia in the wild-type eye (Fig. 1A), the eye with *fruB* overexpression exhibited a broad range of abnormalities: the ommatidium was disrupted in shape, the border between neighboring ommatidia became shallow with a melted appearance, bristles were lost with a few remnants, and the entire compound eye was reduced in size (Fig. 1D). When the fly carried a copy of either *EcR^M554fs^* or *EcR^V559fs^*, null alleles of *EcR*, the effects of overexpressed FruB were markedly mitigated, except for the reduced size of the compound eye (Fig. 1E,F). The *EcR* mutant heterozygosity induced only a moderate roughness of the compound eye (Fig. 1B,C), which cannot explain the observed suppression of FruB-induced eye phenotypes by a mutant copy of *EcR*, suggesting that *EcR* genetically interacts with *fru*, at least when FruB is ectopically expressed in the eye.

### *EcR* increases mAL neurons with the male-specific neurite

Because both *fru* and *EcR* are primarily involved in the control of development, we presumed that these genes cooperate to organize the neural circuitry for male courtship behavior during development. The male-specific FruM proteins are expressed in ∼2000 neurons (Lee et al., 2000; Usui-Aoki et al., 2000), some of which manifest structural sex differences as a result of FruM effects to promote male-typical differentiation (Kimura et al., 2005; Kohl et al., 2013), while others are present only in either sex due to FruM-dependent survival or death (Kimura et al., 2008; Ren et al., 2016). These sex differences at the single neuron level ultimately lead to sexual dimorphisms in neural circuitries and their behavioral outputs (Pavlou and Goodwin, 2013). To unravel the possible roles of EcR for modulating FruM effects to produce sex differences in single neurons, we focused on a particular subset of *fru*-expressing interneurons called mAL, because they display striking sex differences (Cachero et al., 2010; Kimura et al., 2005; Yu et al., 2010), and because we know some of the molecular mechanisms underlying these sex differences (Ito et al., 2012, 2016). mAL neurons exhibit sexual dimorphism in three respects: the number of cells composing the mAL cluster is five in females versus 29 in males; the ipsilateral neurite is not present in any mAL neurons in females, whereas it is present in some mAL neurons in males; and the tip of the contralateral neurite in the subesophageal ganglion bifurcates in females, whereas there is no branching at the tip in males (Kimura et al., 2005). Functionally, mAL neurons represent second-order interneurons in the processing of contact-chemical sex pheromones, and they control alternate wing motion during courtship song generation in males (Cohn et al., 2015; Koganezawa et al., 2010; Kallman et al., 2015).

**Fig. 2.**
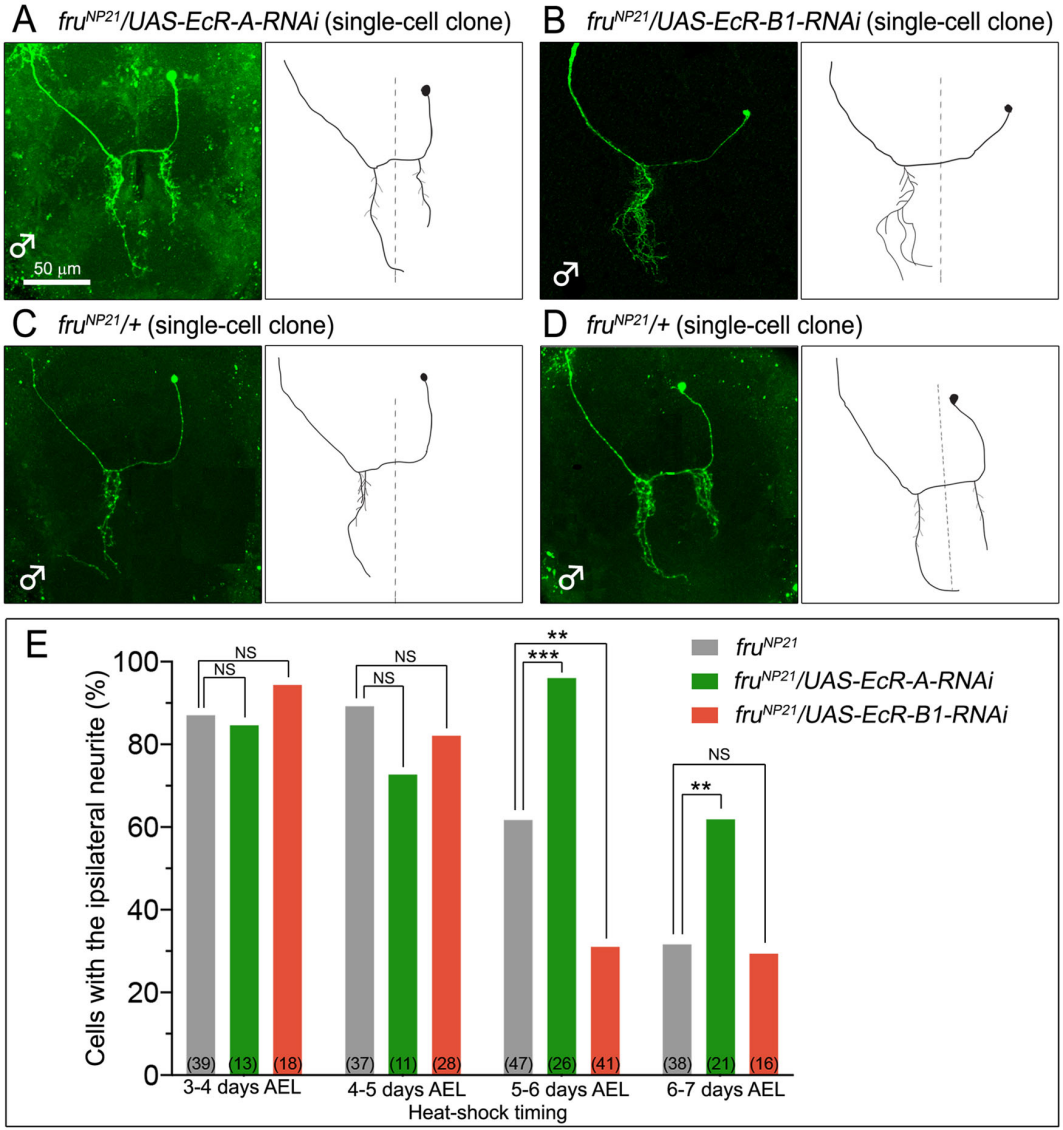
***EcR-A* knockdown increases, whereas *EcR-B1* knockdown decreases the proportion of mAL neurons devoid of the male-specific ipsilateral neurite in the male brain.** (A-D) Single-cell clones of mAL neurons expressing RNAi against the *EcR-A* (A) or *EcR-B1* (B) isoform and respective control clones (C,D) are shown. Drawings of visualized single mAL clones are illustrated in the right-hand column of each image. Scale bar: 50 µm. All clones shown were obtained in flies heat-shocked at 5-6 days AEL. (E) The proportion of mAL neurons with the ipsilateral neurite (ordinate) is compared between the control genotype (*fru^NP21^*/*+*, left-hand bars) and the cells with knockdown (right-hand bars) of *EcR-A* (green) or *EcR-B1* (red) in flies heat-shocked at four different time points as indicated in the abscissa. The number of clones obtained is shown in parentheses. Statistical differences were evaluated by the Fisher's exact test (***P<0.001; **P<0.01; NS, not significant).

To selectively visualize and manipulate mAL neurons, we adopted the Mosaic Analysis with a Repressible Cell Marker (MARCM) technique (Lee and Luo, 1999), in which *fru*-specific GAL4 (expressed via *fru^NP21^-GAL4* in this study) is activated only in cells that stochastically lose a *GAL4*-repressing transgene *GAL80* by chromosomal recombination in response to heat shock-induced Flippase expression (via *hs-FLP*). The GAL4 protein thus produced in a small subset of *fru*-positive cells drives expression from *UAS-mCD8::GFP* for fluorescent marking of the entire structure of these cells as well as expression from *UAS-EcR-RNAi* for knocking down *EcR* in these cells. We used *UAS-EcR-A-RNAi* to knock down the EcR-A isoform and *UAS-EcR-B1-RNAi* to knock down EcR-B1. Both RNAi constructs significantly reduced the respective mRNA expression (Fig. S1). We were unable to obtain a tool for EcR-B2 knockdown, however. We optimized the timing to apply the heat shock for inducing chromosomal recombination so that mAL neurons were predominantly labeled (and manipulated) and the *EcR* knockdown effect was maximized. We found that MARCM neuroblast clones that label all constituent cells of a single mAL cluster are not adequate for this analysis, because practically all neurites of the entire set of mAL neurons from both brain hemispheres overlap one another, making it difficult to observe single neuron structures with no ambiguity. We therefore relied on an analysis with single cell MARCM clones (Fig. 2A-D). We quantified the proportion of mAL neurons with the male-specific ipsilateral neurite (mAL with ILN, ILN[+]) in all mAL single cell clones obtained and used this value as an estimate of the level of masculinization of mAL neurons, based on the knowledge that reductions in functional FruBM result in a small proportion of ILN[+], without producing neurons that have a shorter or longer ILN (Ito et al., 2012). We found that, in control flies, the proportion of ILN[+] varied widely depending on when the heat-shock treatment for clone induction was administered to an animal. The heat-shock treatments at 3-4 days after egg laying (AEL) or 4-5 days AEL invariably yielded a high level of ILN[+] induction, i.e. ∼90% (Fig. 2E). Heat shock applied at 5-6 days AEL also resulted in a high ILN[+] rate, ∼60% (Fig. 2E). In contrast, when heat shock was given at 6-7 days AEL, the proportion of ILN[+] was only ∼30% in control flies (Fig. 2E). This observation is consistent with the notion that the neuroblast produces predominantly ILN[+] during the larval stage, and then generates mainly ILN[–] after pupariation, representing a fate change from ILN[+] to ILN[–] that occurs depending on whether the neuron is born before or after the pupariation. Notably, when EcR-A was knocked down, the proportion of ILN[+] was always high irrespective of the heat-shock timing; the proportion was ∼100% at 5-6 days AEL and ∼60% at 6-7 days AEL (Fig. 2E). Remarkably, EcR-B1 knockdown had a contrasting effect; the proportion of ILN[+] declined to ∼30% for the fly group heat-shocked at 5-6 days AEL (Fig. 2E). We propose that EcR-A and EcR-B1 function in an inverse manner for the fate switching between ILN[+] and ILN[–], which occurs at pupariation.

### EcR forms a complex with FruBM

**Fig. 3.**
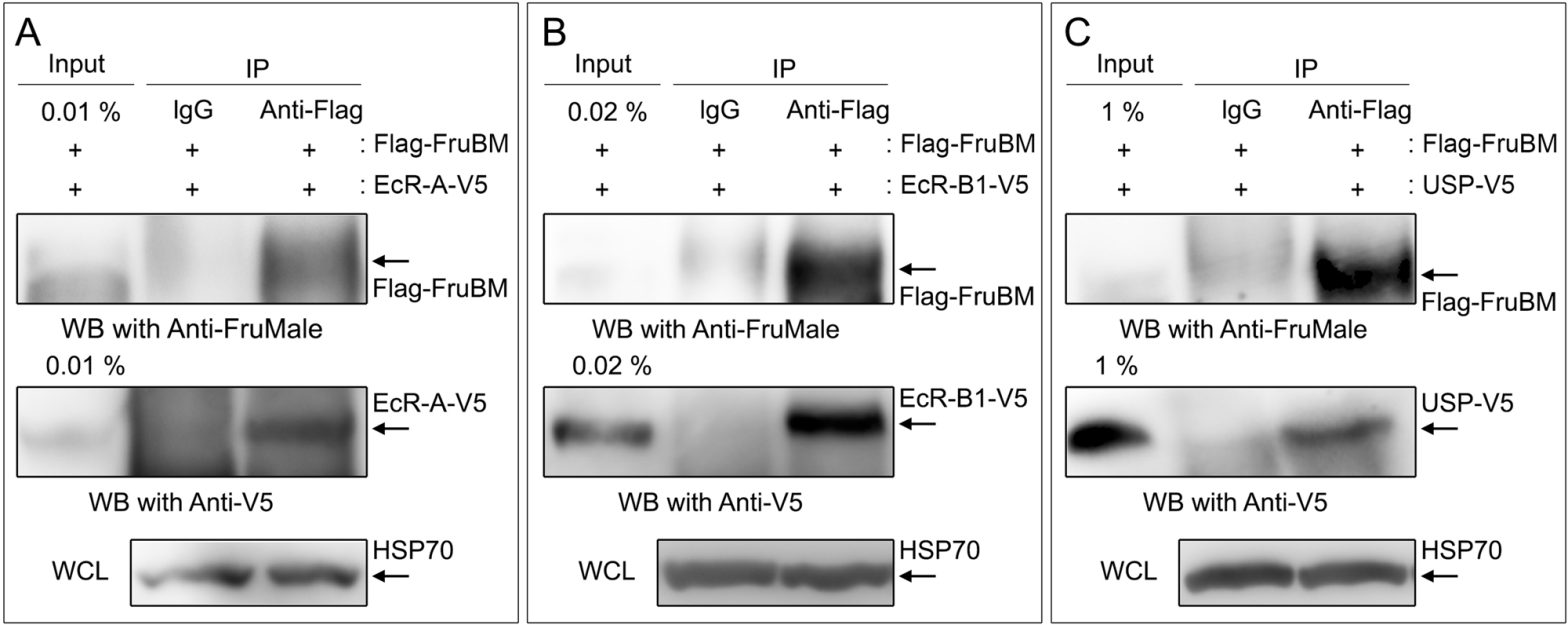
**FruBM forms a complex with EcR-A, EcR-B1 and/or Usp.** (A-C) Lysates of S2 cells cotransfected with a construct encoding Flag-tagged FruBM and that encoding either of V5-tagged EcR-A (A), EcR-B1 (B) or Usp (C) were precipitated with an anti-Flag antibody (IP), followed by western blotting (WB) to detect proteins with an anti-V5 antibody. HSP70 detected in whole cell lysates (WCL) served as a loading control. The band corresponding to FruBM, EcR-A, EcR-B1 or Usp is indicated by arrows.

Next, we attempted to clarify the molecular basis for the EcR action to switch the neural fate from ILN[+] to ILN[−] across pupariation. Because both EcR and FruM likely act through chromatin remodeling (Ito et al., 2012; Sedkov et al., 2003), we tested the possibility that they form a complex to regulate transcription. We transfected the *Drosophila* cell line S2 with constructs that each encoded a tagged version of EcR isoforms and FruBM to obtain cell lysates for coimmunoprecipitation assays. We chose FruBM as the isoform of FruM to test here, because this isoform is the most prevalently expressed and the most potent as a masculinizer (Billeter et al., 2006; Neville et al., 2014; Usui-Aoki et al., 2000; von Phillipsborn et al., 2014). Immunoprecipitation of S2 lysates with an anti-Flag antibody that recognizes FruBM yielded EcR-A, EcR-B1, and an EcR partner, Usp (Yao et al., 1992), in addition to FruBM, as detected by western blotting with an anti-V5 antibody that recognizes EcR isoforms and Usp (Fig. 3). We conclude that the two isoforms of EcR tested (EcR-A and EcR-B1) and Usp form a complex with FruBM.

### Ecdysone regulates transcription of the FruBM target gene *robo1*

**Fig. 4.**
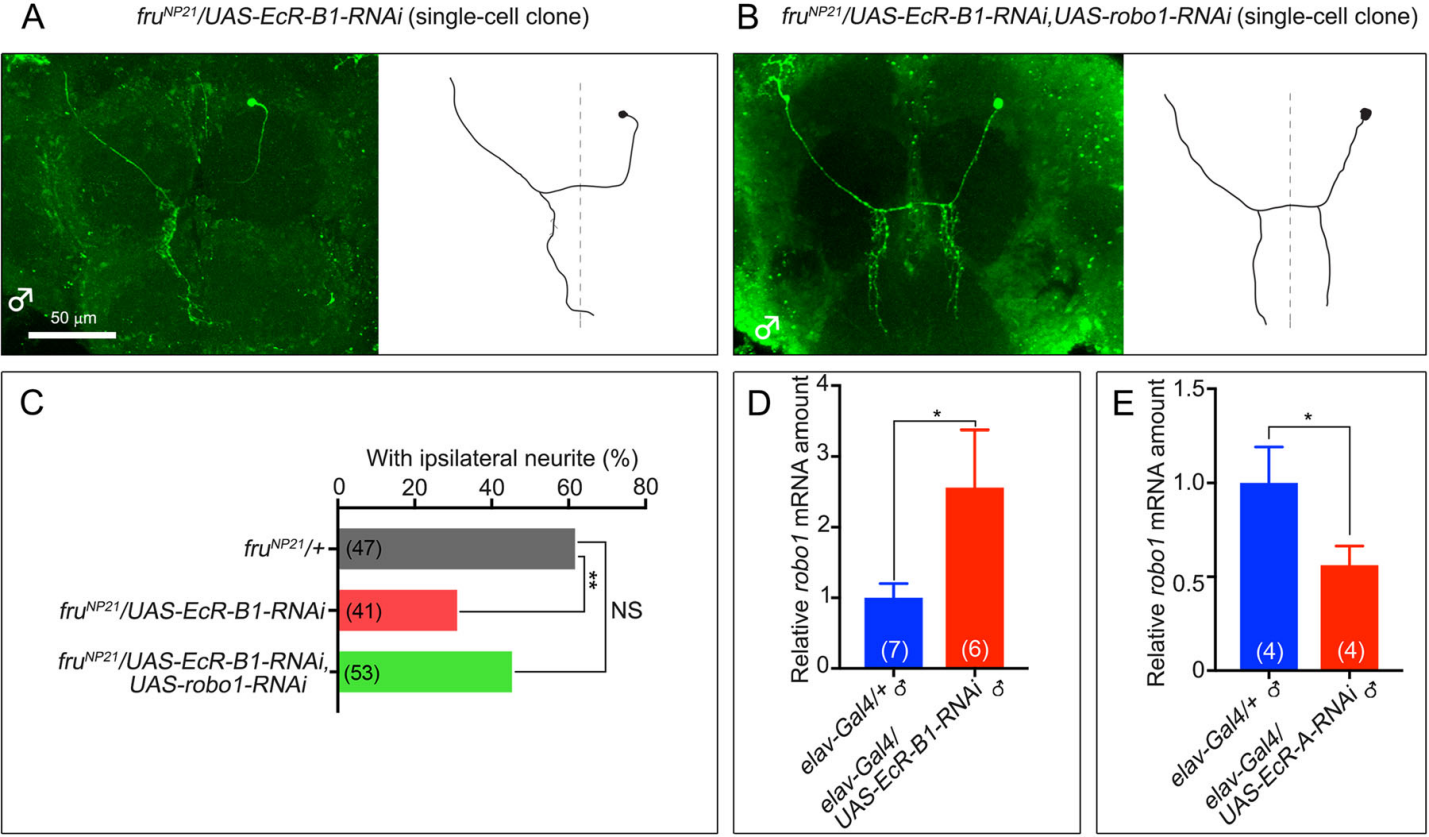
***EcR-B1* promotes whereas *robo1* impedes the ipsilateral neurite formation.** (A,B) Examples of single cell mAL clones without (A) or with (B) the ipsilateral neurites upon knockdown of *EcR-B1* alone (A) or together with *robo1* (B). Heatshock of 37°C for 15-20 min was applied 5-6 days AEL to induce the recombination of chromosomes. Scale bar: 50 µm. (C) The proportion of neurons with the ipsilateral neurite (%, ordinate) is compared for three genotypes as indicated. The number of clones analyzed is shown in parentheses. Statistical significance was evaluated by the Fisher's exact test (**P<0.01; NS, not significant). (D,E) Relative amounts of *robo1* mRNA determined by qPCR were compared between control white pupae (*elav-GAL4/+*) and white pupae in which *EcR-B1* (*elav-GAL4/UAS-EcR-B1-RNAi*; D) or *EcR-A* (*elav-GAL4/UAS-EcR-A-RNAi*; E) was knocked down. Isolated CNSs were used as the source of RNA. The number of replicates each with 10 white pupae is indicated in parentheses. Statistical significance was evaluated by the Student's *t*-test (*P<0.05).

**Fig. 5.**
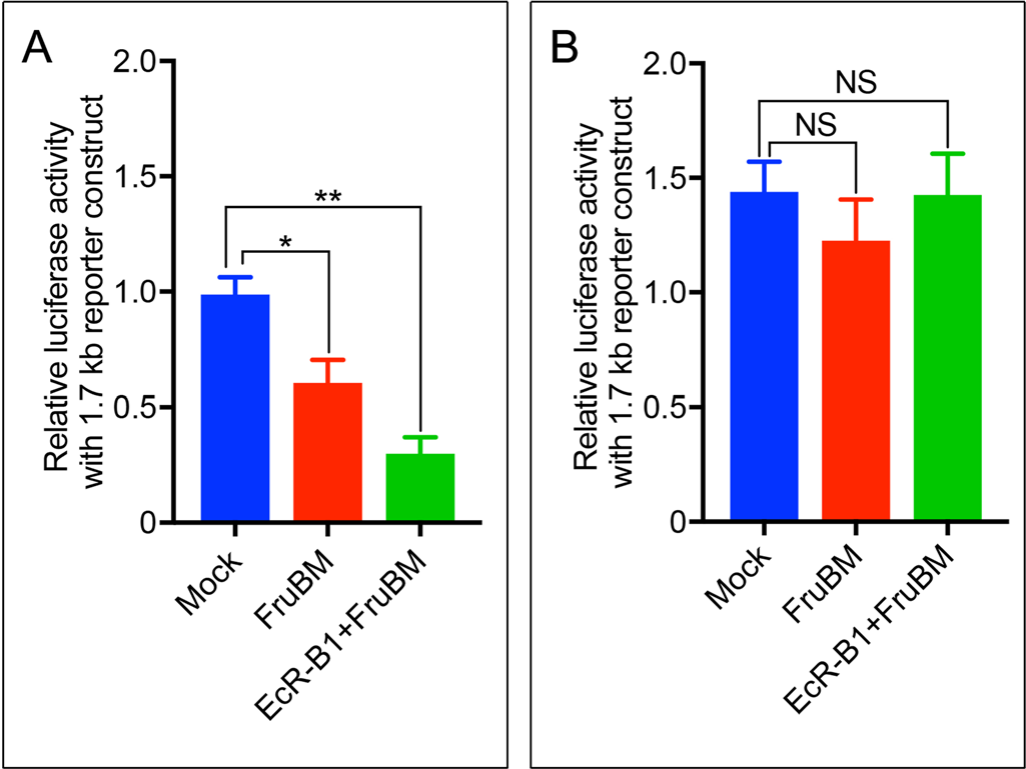
**FruBM-mediated repression of the *robo1* reporter activity is reversed by ecdysone.** (A) *robo1* reporter activities in S2 cells were repressed by *fruBM* transfection (middle bar, FruBM) compared to the control (left-hand bar, Mock), and additional transfection with EcR-B1 enhanced the FruBM-induced repression (right-hand bar, EcR-B1+FruBM) in the absence of ecdysone. (B) Application of 10^−4^ mg/ml ecdysone restored the reporter activity to the control level even in the presence of FruBM, irrespective of whether EcR-B1 was cotransfected or not. Statistical significance was evaluated by the one-way ANOVA with post hoc Tukey's multiple comparison test; **P<0.01; *P<0.05; NS, not significant.

Robo1 is a transmembrane receptor (Kidd et al., 1998) with a key role in determining whether an mAL neuron develops the ILN or not; Robo1 inhibits formation of the ILN in females, whereas *robo1* is transcriptionally repressed by FruBM in males so that the ILN forms in some mAL neurons (Ito et al., 2016). It is tempting to speculate that ecdysone might affect this FruBM action in directing the fate switch from ILN[+] to ILN[−] after pupariation. In keeping with this supposition, *robo1* knockdown impeded the effect of *EcR-B1* knockdown to reduce the proportion of ILN[+] in single cell mAL clones (Fig. 4A-C). This observation at the cellular level *in vivo* is consistent with the idea that EcR-B1 represses *robo1* transcription, thereby promoting ILN formation. To examine this possibility, we quantified *robo1* mRNA by quantitative polymerase chain reaction (qPCR) in white pupae with or without *ECR-B1* knockdown. Indeed, *robo1* mRNA was significantly increased upon *EcR-B1* knockdown (Fig. 4D). Of note, *EcR-A* knockdown had a contrasting effect on *robo1* transcription, i.e. it decreased the level of *robo1* mRNA (Fig. 4E). In view of the fact that EcR forms a complex with FruBM (Fig. 3), it would be conceivable that the effect of EcR on *robo1* transcription is, at least in part, mediated through the EcR-FruBM complex. We thus carried out reporter assays in S2 cells with a *robo1* promoter-luciferase fusion construct that contained a 1.7 kb fragment with the FruBM-binding site (Ito et al., 2016) and its flanking regions. In support of our previous finding (Ito et al., 2016), the reporter transcription was repressed by *fruBM* transfection (Fig. 5A). Additional transfection of S2 cells with EcR-B1 enhanced the repressor activity of FruBM on *robo1* reporter transcription (Fig. 5A). Interestingly, when ecdysone was added to the culture medium, FruBM was unable to repress transcription from the *robo1* promoter (Fig. 5B). In the presence of ecdysone, transfection of *EcR-B1* in addition to *fruBM* did not increase or decrease the *robo1* reporter activity (Fig. 5B). Based on these observations, we suggest that *robo1* transcription is repressed by FruBM, and ecdysone impedes the FruBM repressor action. The fact that EcR-B1 overexpression did not affect the FruBM action in the presence of ecdysone implies that EcR-B1 expressed endogenously in S2 cells is sufficient for mediating the ecdysone action to impede FruBM-induced repression of *robo1* transcription.

## DISCUSSION

The present study unraveled a novel role for EcR. Namely, EcR was found to switch the cell type of a group of *fru*-expressing neurons depending on whether the cells are produced before pupariation when the systemic ecdysone level is low or produced immediately after pupariation when the ecdysone surge occurs. More specifically, we found that mAL neurons with the male-specific ipsilateral neurite (ILN[+]) are preferentially generated before pupariation, whereas those without the ipsilateral neurite (ILN[−]) are generated after pupariation.

The EcR-B1 isoform has been demonstrated to recruit the CREB-binding protein (CBP) with the activity of a histone acetyl transferase (HAT) in the presence of ecdysone, in order to activate transcription of *sox14* via H3K27 acetylation in this locus for facilitating dendrite pruning of sensory neurons during metamorphosis (Kirilly et al., 2011). In contrast, FruBM is known to recruit HDAC1 as mediated by the Transcriptional Intermediary Factor 1 (TIF1) homolog Bonus to its target sites on the genome, presumably resulting in gene silencing for the induction of male-typical development of neurons (Ito et al., 2012). According to a prevalent bimodal switch model, steroid hormone receptors recruit corepressors in the absence of hormone and coactivators in its presence (Johnston et al., 2011; Sedkov et al., 2003). In the present case, ecdysone seems to turn the repressor role of the EcR/FruBM complex off for switching the type of neurons to be produced, i.e. from the ILN[+]-type mAL to ILN[−]-type mAL. A recent study revealed a novel mode of EcR action in the absence of ecdysone, wherein dMi-2 replaces Usp in the complex to induce chromatin remodeling for gene silencing (Kreher et al., 2016). This mechanism may be excluded from possible modes of action of the EcR-FruBM complex, because FruBM invariably coprecipitates with Usp.

**Fig. 6.**
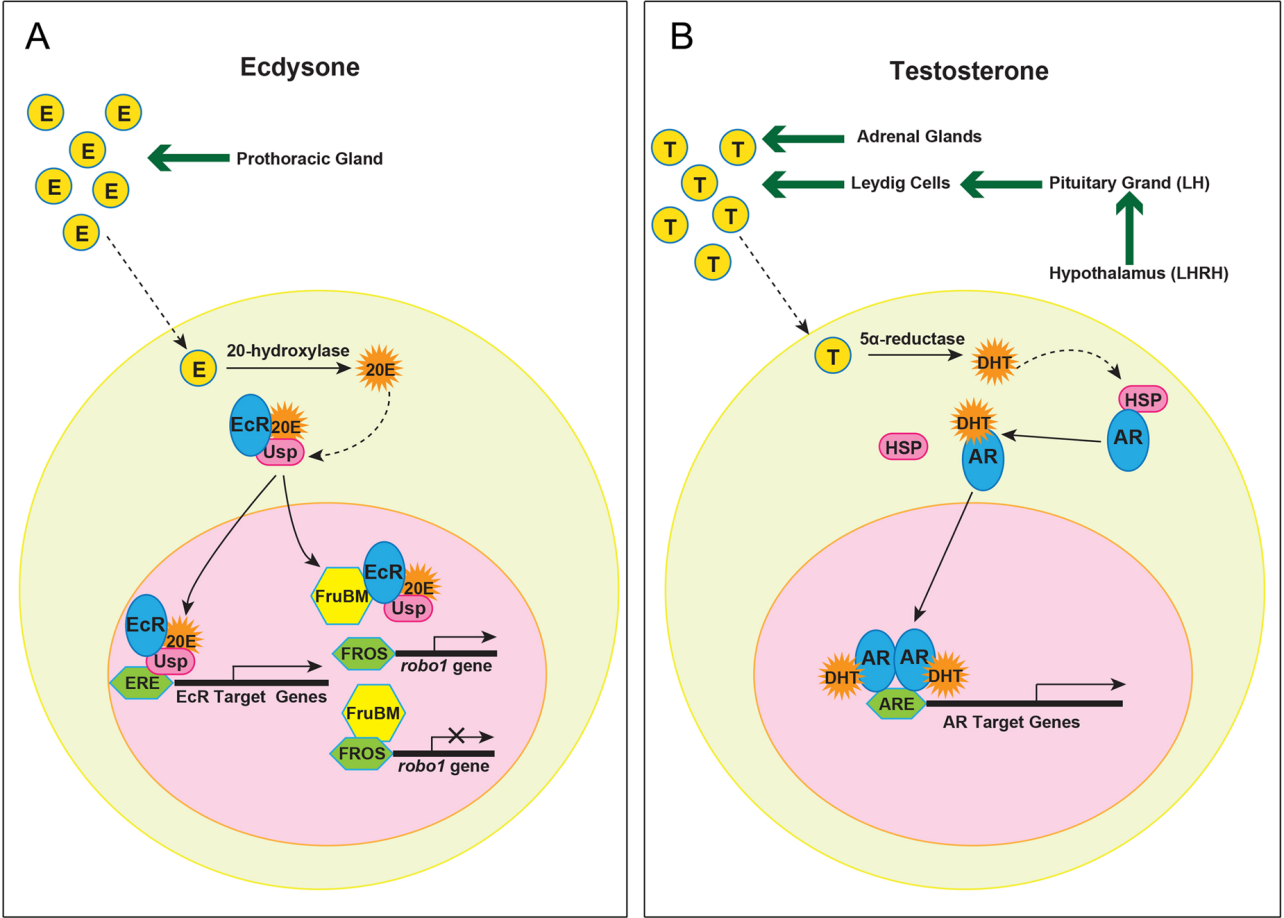
**A model for steroid hormone actions in sex-type specification of a cell.** (A) Ecdysone action in *Drosophila*. (B) Testosterone action in vertebrates.

In this study, we classified male mAL neurons into two groups based solely on the presence or absence of the male-specific ipsilateral neurite. We have previously demonstrated that the male-specific ipsilateral neurite forms when the guidance cue receptor gene *robo1* is transcriptionally repressed (the male state), whereas this neurite does not form when *robo1* is transcriptionally activated (the female state; Ito et al., 2016). FruBM plays a role in switching the *robo1* transcription state; it represses *robo1* transcription in males, while *robo1* is transcribed in females that lack FruBM (Ito et al., 2016). Our result is consistent with the idea that the observed effects of EcR-B1 knockdown on the ipsilateral neurite formation in the male brain are mediated by altered regulation of *robo1* transcription by FruBM. It is plausible that the inclusion of EcR-B1 in the FruBM-containing complex enhances transcriptional repression of *robo1* in the absence of ecdysone, and binding of ecdysone to EcR-B1 results in the restoration of *robo1* transcription (Fig. 6). *robo1* repression in the absence of ecdysone promotes the production of ILN[+], whereas *robo1* activation in the presence of ecdysone promotes that of ILN[−]. Thus, EcR-B1 may function as a fate-controlling switch between ILN[+] and ILN[−], either of which is chosen depending on the ecdysone titer.

It appears that EcR-A has an ability to confer the male-fate on *fru*-expressing neurons other than mAL neurons; decreased EcR-A expression results in a decrease in the volume of male-enlarged glomeruli in the antennal lobe, and a concomitant increase in male-to-male courtship in male flies (Dalton et al., 2009). There is a report describing that female flies of *ecdysoneless^1^* (*ecd^1^*) mutants with a decreased ecdysone titer generate courtship songs that are similar to those produced by males when courted by a male (Ganter et al., 2012). These mutant females were also reported to show a reduction in ovipositor-extrusion attempts toward a courting male to express their unwillingness to mate (Ganter et al., 2012). These observations might suggest that EcR plays a role in neural feminization. Alternatively, the male-like behavior observed in *Ecd^1^* mutant females might result from impairments of sex-specific splicing of sex-determination factor transcripts, because Ecd is a component of the U5 snRPN pre-mRNA splicing complex (Claudius et al., 2014).

Our study showed that EcR is an important component in the FruBM-containing protein complex that instructs certain *fru*-expressing neurons to develop a male-specific structure in the male brain. Thus ecdysone signaling acquires a sex-specific function by crosstalk with a sex-determination pathway component, exhibiting an action comparable to steroid sex hormones in vertebrates on neurons to promote or inhibit the formation of a male-specific structure, depending on the developmental context (Fig. 6).

## MATERIALS AND METHODS

### Fly strains

Flies were reared on cornmeal-yeast medium at 25°C. Canton-S served as a wild-type control. The *EcR^M554fs^* and *EcR^V559fs^* alleles (EMS-induced null alleles; Bender et al., 1997) were generous gifts from Dr T. Kitamoto (University of Iowa, Iowa City, USA). The *UAS-EcR-A*-*RNAi* (BL9328) and *UAS-EcR-B1*-*RNAi* (BL9329) were obtained from the Bloomington Drosophila Stock Center.

### Modifier screens

The female flies with both *GMR-GAL4* and *UAS-fru-typeB^+^* transgenes were crossed with male flies from mutant stocks reported to have developmental defects in the nervous system (FlyBase: http://flybase.bio.indiana.edu/). In this screen, we overexpressed a FruCOM protein rather than a FruM protein when inducing the rough eye phenotype, as the former yielded more viable offspring. The nomenclature for Fru isoforms is adapted from that used in our previous study (Usui-Aoki et al., 2000) and different from that of other groups (Song et al., 2002). Among the 5 Fru C-terminal variants, TypeB was most effective at rescuing the *fru^sat^* mutant phenotype (Billeter et al., 2006; Usui-Aoki et al., 2000; von Philipsborn et al., 2014) and thus was most likely to yield modifiers that were relevant to the *in vivo* functions of *fru*. Images of the compound eye surface were obtained with a scanning electron microscope (SU8000; Hitachi High-Technologies, Tokyo, Japan).

### Coimmunoprecipitation assays

In the coimmunoprecipitation assays for EcR-A, EcR-B1, Usp and FruBM, constructs encoding each protein were overexpressed in S2 cells. Then, 5 µg of one of the pMT-HA-EcR-A-V5-His, pMT-HA-EcR-B1-V5-His or pMT-MYC-Usp-V5-His plasmid vectors and 5 µg of the pMT-FLAG-fruBM plasmid vector were transfected into S2 cells (5×10^7^ cells) using FugeneHD (Roche Diagnostics, Indianapolis, IN, USA), and protein expression was induced by addition of copper sulfate. Lysates were prepared by homogenizing in a cold lysis buffer [50 mM HEPES, pH 7.5, 100 mM NaCl, 50 mM ZnSO_4_, 10 mM NaF, 0.2% NP40 and complete Protease Inhibitor (Roche)] for 1 h at 4°C, then incubated with rabbit IgG (I0500C, Invitrogen) or rabbit anti-Flag antibody (F7425, Sigma-Aldrich) in the aforementioned lysis buffer for 3 h at 4°C. The immuno-complexes were precipitated using Dynabeads^TM^ Protein G (10004D, Invitrogen) according to the manufacturer's instructions. Finally, the immuno-complexes were analyzed by western blotting with a primary antibody, anti-V5 (1:5000; 46-0705, Invitrogen), FruMale (Usui-Aoki et al., 2000), or mouse anti-Hsp70 (1:5000; H5147, Sigma-Aldrich), and, as a secondary antibody, with horseradish peroxidase (HRP)-conjugated, anti-rabbit or mouse IgG antibody (1:3000; Sigma-Aldrich).

### Reporter assays

Reporter assays were carried out with the *robo1* promoter luciferase reporter as described in Ito et al. (2016). The pGL3-promoter vector carrying a 1.7 kb *robo1* promoter fragment was used as a reporter construct. The phRLsv40 *Renilla* luciferase vector (Promega, Fitchburg, USA) served as an internal control. First, 100 ng of a reporter construct and 10 ng of an internal control were co-transfected into S2 cells (2×10^6^ cells) with either pact-HA-FLAG-fruBM or pact-MCS (Ito et al., 2016) using FugeneHD (Roche Diagnostics). Cells were lysed after 48 h of transfection with a passive lysis buffer (Promega), and luciferase activity was measured using a Dual-Luciferase Assay System (Promega). To standardize the transfection efficiency, the reporter luciferase activity of each sample was normalized to the corresponding control *Renilla* luciferase activity: the luciferase activity of a reporter construct was calculated relative to that of an empty pact-MCS plasmid. All experiments were carried out in triplicate; the relative luciferase activities are shown as the means±s.e.m. α-ecdysone (E9004, Sigma-Aldrich) was dissolved in EtOH at a concentration of 1 mg/ml, and the resulting solution served as a stock solution. This solution was added to the culture medium 24 h after plasmid transfection, so that the final concentration of α-ecdysone was 10^−4^ mg/ml. An equal amount of EtOH was added to the medium for a control culture.

### qPCR

qPCR was performed using a LightCycler 1.0 system (Roche). Total RNA was extracted from the CNS of white pupae using an RNeasy Mini Kit (74104, Qiagen). To quantify *robo1* expression levels (Fig. 4D,E), equal amounts of cDNA were synthesized from the extracted RNA using a ReverTra Ace qPCR RT kit (FSQ-101, TOYOBO, Osaka, Japan). Each cDNA was mixed with SYBR Premix Ex Taq II (RR820A, TAKARA, Kusatsu, Japan) and 5 pmol of both forward (5′-CCACGCTCAACTGCAAAGTGGAG-3′) and reverse (5′-AACTGGACGCGGTGCGATTTCTT-3′) primers. *RpL32* (*rp49*) was amplified as an internal control using the primer pair 5′-AGATCGTGAAGAAGCGCACCAAG-3′ (forward) and 5′-CACCAGGAACTTCTTGAATCCGG-3′ (reverse). qPCR was conducted at 95°C for 30 s (initial denaturation), followed by 50 cycles of denaturation at 95°C for 5 s, annealing at 55°C for 30 s and elongation at 72°C for 30 s. Data processing was performed using LightCycler Software Ver. 3.5 (Roche).

### Dissection, immunohistochemistry and imaging of the central nervous system (CNS)

For immunostaining, the CNS of 3-5 day-old files was dissected in cold phosphate buffered saline (PBS) with sharp forceps (Dumont #5). After dissection, the CNS was fixed in 4% paraformaldehyde for 1 h followed by two 30-min washings in 0.2% PBS and Tween 20 (PBT). Then the CNS was kept in blocking buffer containing normal goat serum and 0.2% PBT overnight at 4°C. Immunostaining was performed using a rabbit anti-GFP antibody (at a dilution of 1:500) and a mouse anti-nc82 antibody [Developmental Studies Hybridoma Bank (DSHB), University of Iowa, Iowa City, IA; 1:50 dilution]. Tissues were incubated with the primary antibody for 2 days, then subjected to 20-min washings in 0.2% PBT twice. Alexa Fluor 488 anti-rabbit IgG antibody and Alexa Fluor 546 anti-mouse IgG antibody (Invitrogen; 1:200) were used as secondary antibodies. The CNS was stained for 1 day with the secondary antibody, washed for 30 min in 0.2% PBT twice, and then washed for 30 min with 50% (v/v) glycerol in PBS. Finally, the CNS was mounted on a slide glass with 80% (v/v) glycerol in PBS. Images were acquired with a LSM 510 META confocal microscope (Zeiss, Oberkochen, Germany) using LSM Image Browser software (Zeiss). All images were acquired with either 20× Plan-Apo/0.8 or 40× Plan-Apo/0.95 lenses at a resolution of 512 µm×512 µm with 1 µm intervals.

### Clonal analysis of mAL neurons

We used a *fru^NP21^-GAL4* line to label mAL neurons. The somatic clones were produced using the MARCM method (Lee and Luo, 1999). Flies with the genotype *y hs-flp / Y or w; FRTG13 UAS-mCD8::GFP/ FRTG13 tub-Gal80; fru^NP21^,UAS-Dcr2/+* were used as the control males. The genotype of flies used in clonal *EcR* knockdown experiments was *y hs-flp / Y* (for males) or *w* (females)*; FRTG13 UAS-mCD8::GFP/ FRTG13 tub-Gal80; fru^NP21^/ UAS-EcR-RNAi*. For the production of single-cell clones of mAL neurons, larvae (3-4, 4-5 and 5-6 days AEL) or pupae (6 -7 days AEL) were heat shocked at 37°C for 20 min (larvae) or for 40 min (pupae). Flies to be tested were reared at 29°C after the heat shock in order to enhance the expression of transgenes.

## Supplementary Material

Supplementary information
