# Supplementary material for: Ecdysone signaling regulates specification of neurons with a male-specific neurite in Drosophila
Source: Biol Open. 2018 Feb 15;7(2):bio029744. doi: 10.1242/bio.029744 (PMC5861360; doi:10.1242/bio.029744)
Supplement: Supplementary information [file biolopen-7-029744-s1.pdf]

Supplementary information

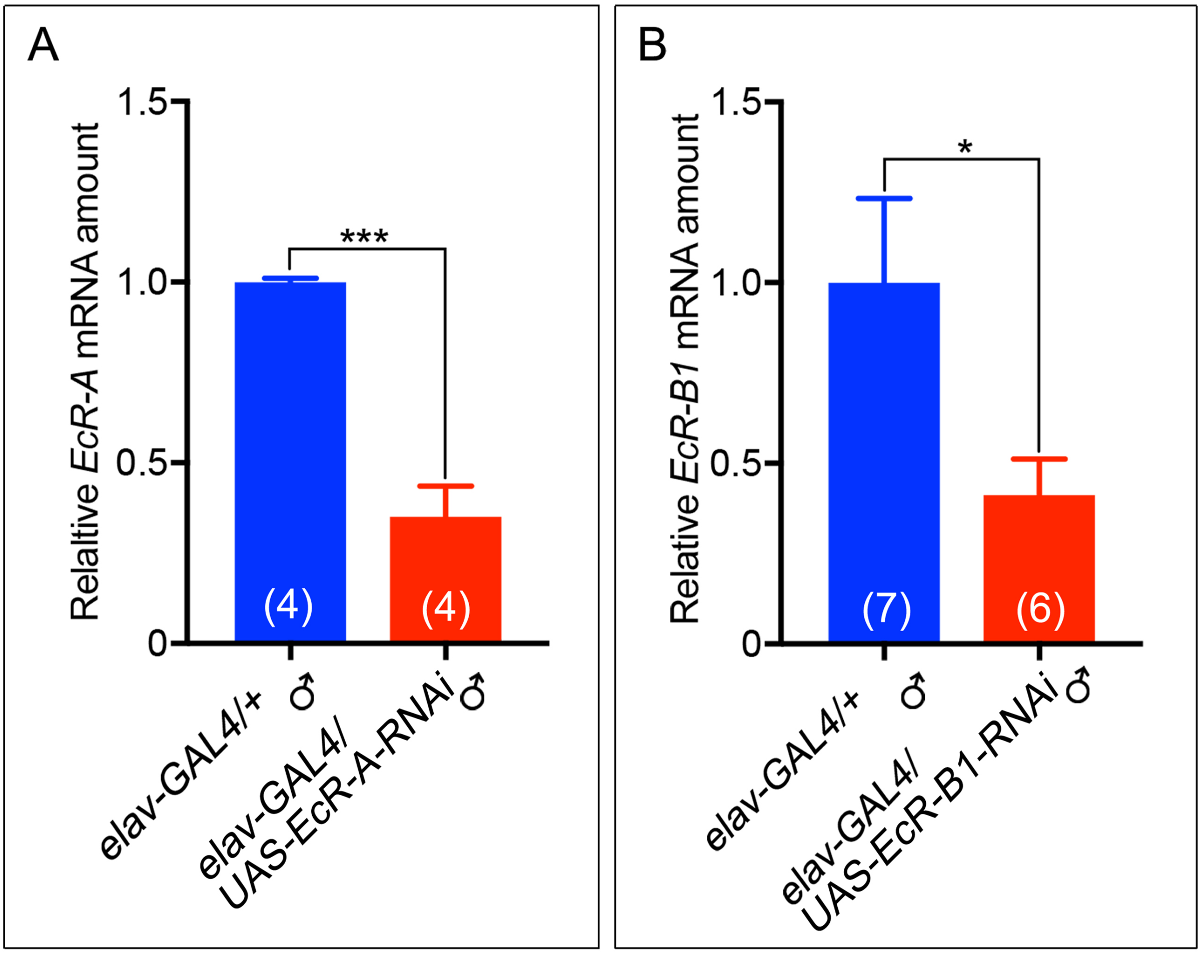

Figure S1. *EcR-A* and *EcR-B1* RNAi constructs effectively reduce the respective mRNA amounts. (**A**, **B**) Relative amounts of mRNA for *EcR-A* (**A**) and *EcR-B1* (**B**) as determined by qPCR were compared between male flies carrying *elav-GAL4* (left-hand side bars) and *elav-GAL4* plus *UAS-EcR-A-RNAi* (**A**) or *UAS-EcR-B1-RNAi* (**B**) (right-hand side bars). The number of replicates each with 10 flies are indicated in parentheses. The statistical significance was evaluated by the Student's *t*-test; ***P<0.001; *P<0.05.

Biology Open • Supplementary information
